# Humic Substances: Determining Potential Molecular Regulatory Processes in Plants

**DOI:** 10.3389/fpls.2018.00263

**Published:** 2018-03-13

**Authors:** Zahid Hussain Shah, Hafiz M. Rehman, Tasneem Akhtar, Hameed Alsamadany, Bahget T. Hamooh, Tahir Mujtaba, Ihsanullah Daur, Yahya Al Zahrani, Hind A. S. Alzahrani, Shawkat Ali, Seung H. Yang, Gyuhwa Chung

**Affiliations:** ^1^Department of Arid Land Agriculture, King Abdulaziz University, Jeddah, Saudi Arabia; ^2^Department of Electronics and Biomedical Engineering, Chonnam National University, Gwangju, South Korea; ^3^Department of Biological Sciences, King Abdulaziz University, Jeddah, Saudi Arabia; ^4^Plant and Forest Biotechnology Umeå, Plant Science Centre, Swedish University of Agriculture Sciences, Umeå, Sweden; ^5^Department of Biology, College of Science, Imam Abdulrahman bin Faisal University, Dammam, Saudi Arabia; ^6^Kentville Research and Development Centre, Agriculture and Agri-Food Canada, Kentville, NS, Canada

**Keywords:** humic acid, epigenetic modifications, genotoxicity, hormonal regulations, nod factors, signaling crosstalk

## Abstract

Humic substances (HSs) have considerable effects on soil fertility and crop productivity owing to their unique physiochemical and biochemical properties, and play a vital role in establishing biotic and abiotic interactions within the plant rhizosphere. A comprehensive understanding of the mode of action and tissue distribution of HS is, however, required, as this knowledge could be useful for devising advanced rhizospheric management practices. These substances trigger various molecular processes in plant cells, and can strengthen the plant’s tolerance to various kinds of abiotic stresses. HS manifest their effects in cells through genetic, post-transcriptional, and post-translational modifications of signaling entities that trigger different molecular, biochemical, and physiological processes. Understanding of such fundamental mechanisms will provide a better perspective for defining the cues and signaling crosstalk of HS that mediate various metabolic and hormonal networks operating in plant systems. Various regulatory activities and distribution strategies of HS have been discussed in this review.

## Introduction

Approximately 60% of soil organic matter consists of humic substances (HSs), which, as key components of the terrestrial ecosystem, are responsible for several complex chemical reactions in the soil (Trevisan et al., 2010). HS are supramolecular structures of heterogenous molecules comprising of sugar, fatty acids, polypeptides, aliphatic chains, and aromatic rings, held together by hydrophobic interactions (such as van der Waals, π-π, ion-dipole) and hydrogen bonds (Piccolo, 2001; Trevisan et al., 2010). HS have been classified into humin, humic acid and fulvic acid, based on different solubility at acid and alkaline pH (Trevisan et al., 2010). This classification is based only on superficial criteria, and does not indicate chemical behavior or provide insights into chemical structures.

Humus is main fraction of humic substances, and is typically prepared by successive steps of fermentation of garbage, agricultural by-products, fresh organic matter from plants, animals, and coal. Fermentation of these substances occurs via activity of soil microbiota under selected physio-molecular conditions such as controlled temperature, time, water, and aeration (Canellas and Olivares, 2014). Regardless of the source of origin of the carbon (i.e., C3 and C4), HS and humic acids extracted from the soil are structurally similar (Muscolo et al., 2013; García et al., 2016a). However, HS have wide and extensive functions in plants (Canellas et al., 2010; Canellas and Olivares, 2014). For example, many studies have shown that plants growing in soils with adequate HS content, or those exposed to foliar spray consisting of HS, are healthier; these plants cope well under stressful conditions, and produce high yields with enhanced nutritional quality. The most convincing demonstration of the effect of HS has been the report of their role in lateral root development and root hair formation (Ramos et al., 2015), which was evaluated by Khristeva (1957), a pioneer in this field of research. Moreover, several studies highlight various physiochemical and biological properties of HS to explain their significance in soil fertility (Hirel et al., 2007; Brown et al., 2014). However, considerable gaps still exist in our understanding of how HS induce signaling in plants that allows them to cope with heavy metal genotoxicity (Yigider et al., 2016), epigenetic modifications (Erturk et al., 2015), and building rhizospheres and microbial interactions (Gao et al., 2015). Moreover, the unique molecular structure of HS and their relative response to antioxidant, drought, and salt stresses has been reported to explain the biological effect of HS on plants (Canellas and Olivares, 2014); one such mechanism involves binding of HS with inorganic trace mineral elements that can then be utilized by various crop plants to trigger potential molecular processes. HS thus function as important ion exchange and metal complexing (chelating) molecules (Beiraghi et al., 2014). In this manner, HS can readily enter plant roots, stems, and leaves, and carry trace minerals from plant surfaces into plant tissues; for this reason, HS are used as key ingredients in high-quality foliar fertilizers (García et al., 2016a).

Similarly, HS, particularly those with a low molecular mass, are readily taken up by plants, and help promote nutrient uptake (Garcia-Mina et al., 2004), or plant growth and development, by acting as hormone-like substances (Vaughan and Malcom, 1985; Nardi et al., 2000, 2002). Likewise, HS also have a positive effect on other plant physiological processes such as leaf chlorophyll content (Vaughan and Malcom, 1985); however, the relationship between physio-molecular activity and biological activity of HS, because of their complex nature, is not as yet completely understood. These scientific highlights related to HS need to be linked with molecular function and reactivity, in order to gain a comprehensive understanding of their role in the functional and regulatory processes of plants.

This review specifically considers the actions of HS on various functional and regulatory processes of plants (e.g., signaling, mineral uptake, abiotic stress tolerance, mitigation of heavy metal genotoxicity, microbial activity, transport, etc.), involving both, their physiological and molecular impacts. The aim of this review is to highlight the roles of HS as an organic biostimulant and its physio-molecular aspects related to plant health.

### Humic Substances and Improved Mineral Nutrition

The presence of HS in soil stimulates the growth of root and shoot by improving mineral nutrition beneath the soil surface. The activity of these substances can be evaluated in terms of the yield and active growth of plants (Zandonadi et al., 2016). HS regulate plant growth and mineral assimilation through their complementary and potentially diverse effects. These effects are generally categorized as direct and indirect (Vaughan and Malcom, 1985; Zandonadi et al., 2013). Activities of HS are primarily dependent upon structural features, functional groups, and their tendency to interact with inorganic and organic ions and molecules residing in the soil substrate (Garcia-Mina et al., 2004). Moreover, HS mainly affect nutrient bioavailability via their ability to form complexes with metallic ions, which enhances the availability of micronutrients (zinc, manganese, copper, and iron); and macronutrients (phosphorus), and particularly when these nutrients are scarce in the soil (García et al., 2016b). Conversely, direct action of HS is related to their localized targeted and non-targeted effects at plant cell membranes, that can initiate biochemical and molecular processes at post-transcriptional levels in roots and shoots (Van Oosten et al., 2017). Generally, targeted effects of HS tend to enhance the uptake of macronutrients and micronutrients in plants. Vaughan and Malcom (1985) used 14-C labeled HS, and found that a minor fraction of these substances, specifically those with lower molecular weight, enter into the root apoplastic pathway. Hence, this feature can regulate the action of HS in promoting the uptake of nutrients by molecular systems and signaling pathways present in root cell membranes (Asli and Neumann, 2010; García et al., 2012; Nardi et al., 2016). Until now, the complete benefit of this type of direct effect is not yet known. It can however be speculated that non-specific action of HS on the leaf or root surface can alter molecular and biochemical processes by regulating the events at both transcriptional and post-transcriptional levels.

### Humic Substances and Abiotic Stress Regulation

Mitigating the effects of abiotic stresses on the growth and development of plants during ontogenesis can significantly benefit the productivity of crop plants. Current knowledge about the controlling mechanisms and strategies is limited (Van Oosten et al., 2017). Optimization of plant growth conditions and provision of water, nutrients, and plant growth regulators can help in preventing abiotic stresses (García et al., 2014). In addition to these conventional strategies, biostimulants like HS are frequently being integrated into production systems with the objective of regulating physiological events in plants to increase the productivity. HS derived from natural materials have gained increasing use by scientists in the last two decades (Yakhin et al., 2016). Moreover, HS provide novel approaches for modifying physiological activities in plants to improve plant growth and resistance toward abiotic stresses.

Various examples indicate the capability of HS to increase abiotic stress tolerance in plants. HS treatment of bent grass (*Agrostis palustris*) and fescue (*Festuca arundinacea*) increased leaf hydration, antioxidant ability, root growth, and shoot growth under dry soil conditions (Zhang and Ervin, 2008; Van Oosten et al., 2017). Recent studies have revealed that fouling-mediated transient blockage of cell wall pores is responsible for the effects caused by HS, which could potentially be associated with a transient mild water stress (a beneficial stress, ‘eustress’) and is responsible for downstream HS-mediated effects on hormonal signaling pathways and plant development (Olaetxea et al., 2015). Integrated application of HS and seaweed extract increases drought tolerance, as well as endogenous levels of cytokinins (Zhang and Ervin, 2008). Similarly, augmentation of humic acid with phosphorus deceased sodium content in bell pepper (*Capsicum annuum* L. ‘Demre’), but increased levels of nitrogen, phosphorus, potassium, iron, magnesium, sulfur, manganese, and copper contents in roots and shoots, which are indicators of the protective effect of HS against salinity stress (Çimrin et al., 2010). Furthermore, Aydin et al. (2012) observed that HS application under saline conditions increased proline content, and reduced membrane leakage and reactive oxygen species (ROS) generation in the common bean (*Phaseolus vulgaris* L.), reflecting better adaptability to saline conditions. Similar to their beneficial effects on field crop dicots, HS are also equally beneficial to field crop monocots (Van Oosten et al., 2017*)*. Vermi-compost applications to rice (*Oryza sativa* L.) dynamically increased the concentration of ROS scavenging enzymes and triggered the activity of anti-oxidative enzymes. These enzymes play a crucial role for inactivation of oxygen free radicals generated in plants under drought and salinity stress (García et al., 2012). Moreover, HS differentially regulate proton ATPases located in vacuolar and cell membranes that ultimately mitigate the hazardous effects of ROS. Correspondingly, when tomato plants were subjected to vermi-compost, the extrusion of protons from the plasma membrane was exceeded by 40%, which increased acid formation and nutrient uptake tendency (Zandonadi et al., 2016). Interestingly, a decrease in proton exclusion was observed in an auxin insensitive mutant of tomato, and indicates that HS may trigger root growth by regulating auxin signaling (Zandonadi et al., 2016). Relatively few studies have explained the physiological effect of HS on the molecular aspects of crop plants. For example, in maize, an isoform of H^+^-ATPase MHA2 gene works as a specific auxin target, and a phospholipase A2 gene (Russell et al., 2006; Canellas et al., 2010, 2011; Pizzeghello et al., 2012) acts as a component of auxin-dependent signaling.

### Prevention of Heavy Metal Genotoxicity and Genetic Instability

Humic substances mitigate the effects of surplus heavy metals that can trigger genotoxicity and genetic instability. Although heavy metals play a vital role as essential micronutrients in several physiological processes of plants (i.e., respiration, photosynthesis, and protein synthesis) by modulating the biological mechanisms of various proteins and enzymes (Erturk et al., 2015), they can nonetheless cause toxicity under extremely high concentrations (Nardi et al., 2007; Aguirre et al., 2009). Recent reports have shown various toxic effects of heavy metals on several plant metabolic processes.

Several heavy metals are mutagenic elements, and their genotoxicity has been demonstrated in various mutagenic assays (Doroftei et al., 2010; Erturk et al., 2012a,b). The synthesis of ROS (i.e., 1O_2_, O^2-^, OH^-^, and H_2_O_2_) might increase the genotoxic effects of these metals (Li et al., 2010), since ROS destroy proteins, nucleic acids, and lipids in a pervasive manner (Erturk et al., 2015). The protective role of HS is primarily related to their association with glutathione biosynthesis, which protects DNA and other cellular entities from the oxidative damage of free radicals. Many authors have reported that the toxic effects of heavy metals can likely be ameliorated by HS (Haghighi et al., 2010), because HS function as antitoxic, anticlastogenic, and antimutagenic agents (Marova et al., 2011). Likewise, a protective effect of HA against dicamba-induced genotoxicity and DNA modification in *Phaseolus vulgaris* L. has been reported by Yildirim et al. (2014). To date, no comprehensive reports exist regarding the retrotransposonal changes caused by heavy metals and DNA mutations in plants. Therefore, investigation of the polymorphic (insertion) role of retroelements and genomic instability in crops under heavy metal stress, coupled with the effects of HA on these polymorphisms, is needed.

Humic substance establish transcriptional interactions with biochemical components and signaling pathways, eliciting dynamic signaling crosstalk inside the plant to cope with various types of stresses (García et al., 2016a). For example, epigenetic modifications such as methylation, alkylation, oxidation, DNA strand breakage, and cross linkage in proteins take place due to the adverse effects of oxidative damage caused by heavy metals (Guangyuan et al., 2007; Erturk et al., 2015). Although plants have evolved various antioxidant defense mechanisms to counter such damage (Apel and Hirt, 2004; Madsen-Bouterse et al., 2010), heavy metals trigger various epigenetic mechanisms, including DNA methylation, histone modification, and the expression of non-coding RNAs (Erturk et al., 2015) that regulate gene expression in multiple ways, especially under stress conditions (Cheng et al., 2012). It has been reported that gene expression has significant correlations with epigenetic modifications such as DNA hypo- and hyper-methylation; likewise, various biological pathways (i.e., transcriptional gene silencing and transposable element inactivation) are also linked with phylogenetic developments (Cheng et al., 2012; Büyükkeskin et al., 2014).

Humic substances are thought to play a role in restricting retrotransposon activity, limiting excessive genomic changes (Picault et al., 2009). Plants have complex genomes with large amounts of inactive transposable elements (TEs) and a few trans-positionally active TEs (Casacuberta and Gonzalez, 2013). Various stresses, including those caused by heavy metals, are responsible for increasing retrotransposon activity (Picault et al., 2009). Although TE-generated mutations are vital for environmental adaption, active transposable elements might cause potentially harmful genetic modifications and mutations, leading to genomic instability (Casacuberta and Gonzalez, 2013). Therefore, plants have also evolved processes that can suppress TE activity (Pecinka et al., 2010). Yigider et al. (2016) reported that Mn exhibits a dynamic role in inducing epigenetic changes in *Zea mays*; however, its effect is countered by HA treatments at different doses. In fact, the protective action of HS is associated with their antioxidant and enzyme-regulatory actions, which are reflected in the reduced expression of TEs (Picault et al., 2009; Yigider et al., 2016). HS augment the tolerance of plants to heavy metal toxicity (**Table 1**) by coordinating the actions of non-enzymatic antioxidants and ROS-detoxification enzymes.

**Table 1:** Humic substance (HS)-triggered mode of action and alterations that regulate specific activities in plants to mitigate stresses.

Mechanisms	Plants	Alterations	Functions	Reference
Photosynthesis and energy metabolism	*Agrostis tenuis, Arabis paniculata, Lonicera japonica, Pteris vittata*	Abundance of photosynthetic proteins of the cytochrome b6/f complex, OEC, RuBisCO, ATPase in green tissues	Detoxification of heavy metals	Visioli and Marmiroli, 2013
Protein and nitrogen metabolism	*Alyssum lesbiacum, A. paniculata, B. juncea, P. americana*	Modulation and induction of specific proteins	Detoxification of heavy metals and prevention against biotic stress	Bona et al., 2011; García et al., 2016b
Sulfur sequestration	*Alyssum lesbiacum, A. thaliana, Brassica napus*	Modulation of proteins	Detoxification of heavy metals and ROS scavenging	Schneider et al., 2013
Defense protein triggering	*Alyssum bertolonii, N. caerulescens, P. vittata*	Disruption of the cellular redox status	Detoxification of heavy metals, ROS scavenging, abiotic and biotic stress tolerance	DalCorso et al., 2013
Membrane trafficking	*A. thaliana, A. halleri, N. caerulescens*	Activation of ATPase and ionic pumps residing in membrane	Sequestration of metal ions into vacuoles and other sub-cellular compartments, and their export across the membranes	DalCorso et al., 2013; Schneider et al., 2013
Metal ligation	*A. lesbiacum, N. caerulescen, P. americana*	Abundance of small cysteine-rich metal-binding proteins and constitutive expression of respective genes	Detoxification of heavy metals	Bona et al., 2011; Ramos et al., 2015

### Humic Substances: Signaling, Regulatory, Antioxidant, and Physiological Roles in Roots

The biological activity of HS has been extensively investigated for many years, but comprehensive and uniform knowledge of HS activity still needs to be integrated into a single model (Mora et al., 2014). The extant results indicate that HS trigger effect-oriented action via involvement of different but integrated mechanisms, which function as sequential events of complex networks at transcriptional and post-transcriptional levels (Garcia-Mina et al., 2004; Mora et al., 2014). HS exhibit direct effect-mediated actions, such as the interaction of rhizospheric HS with plant roots. Despite some reports on the possible penetration of the HS fraction into the root apoplast and epidermal cell layer, conclusive results have not yet been obtained (Mora et al., 2014). However, some biological effects of HS on plant activities (**Table 1**) are obvious, as their physical accumulation cannot be ruled out in plant root and shoot systems due to their reported presence on the root surface (Olaetxea et al., 2015). Moreover, HS play a significant role in mediating signaling, regulatory, and physiological events in roots.

Recent studies have also described the vital role of increased root hydraulic conductance, triggered by abscisic acid (ABA) and aquaporin gene expression, during shoot growth enhancement caused by applications of sedimentary humic acid (SHA) to roots (García et al., 2014; Olaetxea et al., 2015). However, data on the role of HS-mediated increase in ROS in root and shoot systems are yet to be obtained (García et al., 2016a). In general, these findings suggest that interconnected signaling pathways and physiological events play an essential role in shoot growth processes, stimulated by humic acid with sedimentary origin (Mora et al., 2012). Micromorphological and macromorphological effects are identified as two prominent and direct effects of HS on root development (Trevisan et al., 2010, 2011; Mora et al., 2012). Micromorphological effects are manifested by an increase in absorbent hairs and lateral roots, whereas macromorphological effects are observed as an increase in root dry weight, secondary root number, and primary and secondary root thickness. Many reports identify the HA derived from vegetal residues as a root-proliferation enhancer at later developmental stages, which modifies the root architecture (Canellas and Olivares, 2014). This is further linked with activation of root PM – H^+^-ATPase activity, via auxin [indole acetic acid (IAA)] activity and possibly through NO (nitric oxide)-dependent pathways (Canellas et al., 2002; Zandonadi et al., 2010).

Genes encoding different metabolic pathways responsible for nutrient uptake and upregulation of auxin biosynthesis pathways, help mediate the effects in the roots of corn, tomato, and *Arabidopsis* (Zandonadi et al., 2010; Ramos et al., 2015). The superstructure organization of HA helps explain their effects. For example, various reports explain both the presence and activity of HA in rhizosphere, where it releases structural fragments or molecules to accelerate auxin bioactivity (Canellas and Olivares, 2014). Low concentrations of several hormones have been clearly observed in composted fresh vegetal material (Olaetxea et al., 2015). Various studies have also reported that root development is affected by the activity of relevant phytoregulators that are mediated by the presence of HS near the root system (Jannin et al., 2012; Mora et al., 2014; Olaetxea et al., 2015).

Moreover, HS, in association with signaling agents and their accessory entities, regulate various processes within plant cells, as shown in **Table 2**. These data consequently point toward the potential role of other factors in HS-mediated thickening of roots, increase in dry weight, and secondary root development (Olaetxea et al., 2015; García et al., 2016b). For example, these results highlight scenarios where hormone (auxin and ETH)-dependent pathways could not explain the effect on root structure of the water-soluble fraction of peat-derived HS (Trevisan et al., 2010; Zandonadi et al., 2010). Analytical studies on phytoregulators in HS found that the concentrations of these compounds were under the detection limit (Pizzeghello et al., 2012). Thus, reports that HS hormones or hormone-like structural domains are present in the HA superstructure, particularly during the interaction of HS with cell walls, can only be considered as a viable (and likely testable) hypothesis at this point.

**Table 2:** Humic substance associated signaling activities in plants.

Signaling agents	Accessory entities	Regulatory processes	Indicators	Reference
Auxins	Auxin-responsive promoters (*DR5: uidA* and *BA3: uidA, ABP1*, and *TMK*)	Alter root morphology, by increasing the root surface through auxin-independent signaling pathways	IAA, ETH, or NO	Mora et al., 2012
ROS	Activity of the enzymes peroxidase (POX), ascorbate peroxidase (APOX), catalase (CAT), and superoxide dismutase (SOD)	Regulation of oxidative metabolism, as well as increasing the levels of H_2_O_2_ and decreasing lipid peroxidation, possibly via the transformation and control exercised by enzymes	IAA, NO, and ETH	García et al., 2014, 2016a
ABA	H_2_O_2_ production and Ca^2+^ channel signaling, plasma membrane intrinsic protein (PIP)	Regulation of root hydraulic conductivity, in the shoot growth promoting action and plant development	OsTIP1;2	Olaetxea et al., 2015
*Aquaporins*	Genes of the tonoplast intrinsic aquaporin (TIP) subfamily	Osmoregulation and water flow through tonoplasts	IAA, NO	Ramos et al., 2015
Ca^2+^ gradient	NADPH oxidase, calcium-dependent protein kinase (CPDK)	Regulation of other important processes, including anti-stress regulation and hormone signaling, and cell wall acidification	OsCPK7, OsCPK17	García et al., 2016a

Frequently, ROS and Ca^2+^ signals work collectively in association with protein kinases and protein phosphorylation to regulate post-translational events (Shabala et al., 2016). Moreover, the Ca^2+^-kinase signaling route plays a vital role in elucidating the targeted effects of HS in root development (Ramos et al., 2015). HS-activated signaling pathways are identified at the post-transcriptional level during calcium-dependent protein phosphorylation (Ramos et al., 2015). The process of protein phosphorylation and the role of Ca^2+^-dependent protein kinase activity are reported to be compatible with the pathways in the post-transcriptional expression of HS activity (Ramos et al., 2015; Shabala et al., 2016).

The internal region of the cell remains inaccessible to most HS. However, HS can positively affect the plasma membrane (PM) H^+^-ATPase-driven enzyme expression and proton gradients, and thus can hypothetically serve as a useful biological marker (Cordeiro et al., 2011a,b). It is speculated that root PM receptors sense HS to produce IAA, which not only activate transcription factors and protein metabolism, but also modulate the activity of PM H^+^-ATPase to trigger cell division and growth. Moreover, in maize roots, a high uptake of nitrogen is associated with the upregulation of the transcripts of PM H^+^-ATPase and NO_2_ transporters (Cordeiro et al., 2011b). Canellas et al. (2002) have reported that the mitotic precursors possess twofold higher concentrations of PM H^+^-ATPase in the lateral roots of maize, compared to the control. Quaggiotti et al. (2004) similarly reported eightfold higher concentrations of mRNA (MHA2 transcripts) in lateral roots of maize, compared with the control. Humus interferes with the physiology and biochemistry of plants, putatively due to its accessorial contents like hormones, enzymes, and transporters (García et al., 2014).

**FIGURE 1:**
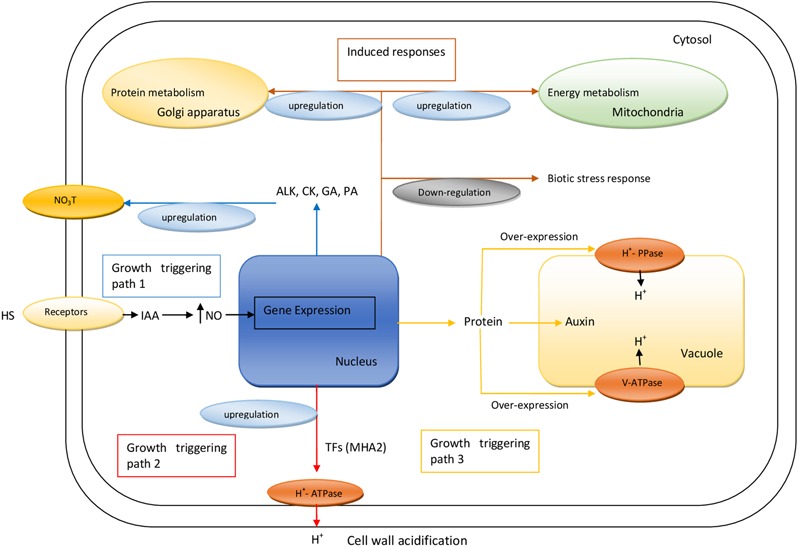
Proposed model illustrating three different growth triggering pathways regulated by HS in root cells of plants. HS, humic substances; IAA, indole acetic acid; ALKs, alkamoids; CK, cytokinin; GA, gibberellic acid; PA, polyamines; V-PPase, vacuolar pyrophosphatase.

Receptors in the cell membrane sense HS in the rhizosphere to produce IAA, which increases the concentration of NO in root cells. Moreover, NO modulates the gene expression to elicit three growth-triggering pathways. The first pathway activates the generation of alkaloids (ALK), cytokinin (CK), gibberellic acid (GA), and polyamines (PA), which upregulate the nitrate transporter (NO_3_T) to maximize nitrogen assimilation. The second pathway upregulates the activity of PM H^+^-ATPase that pumps out H^+^ into the cell wall, causing its acidification and loosening. The third pathway generates proteins whose over-expression upregulates the tonoplast enzymes, V-ATPase and H^+^-PPase, to initiate the influx of auxin and H^+^ into the vacuole. Moreover, other processes such as energy metabolism, protein metabolism, and biotic responses are also triggered in root cells by HS (**Figure 1**).

### Humic Substances: Signaling Crosstalk and Root–Shoot Physiological Events

**FIGURE 2:**
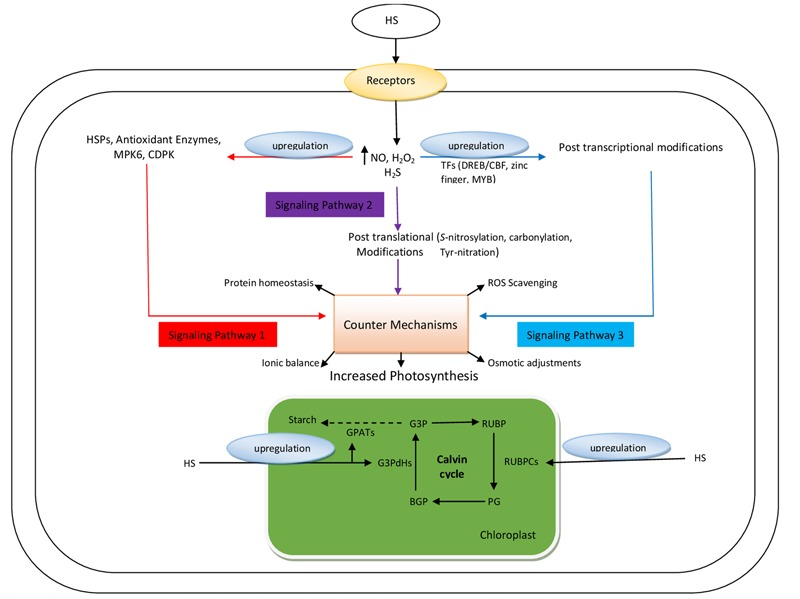
Proposed model illustrating different signaling pathways triggered by HS to increase the photosynthesis activity in plant cells. TFs, transcription factors; DREB, dehydration responsive element-binding factor; CBF, C-repeat binding factor; HSPs, heat shock proteins; MPK6, mitogen activated protein kinase 6; CDPK, calcium dependent protein kinase; G3P, glyceraldehyde-3-phosphate; RUBP, ribulose biphosphate; PG, phosphate glycerate; BPG, biphosphateglycerate; RUBPCs, ribulosoebiphosphate carboxylase; G3PdHs, glyceraldehyde-3-phosphate dehydrogenase; GPATs, glucose-1-phosphate adenylyltransferase.

Several studies have authenticated the ability of HS to regulate root and shoot physiological events. However, the mechanisms of HS action remain unknown (Olaetxea et al., 2016). Various signaling entities such as ABA, nitrate, NO, ROS, and IAA dynamically regulate the root and shoot physiological events by triggering several pathways in response to HS, as shown in **Figure 2**. The various signaling and regulatory components of HS-mediated crosstalk are described in the following sub-sections.

Humic acid accelerates shoot growth via various processes in the root and shoot, such as PM H^+^-ATPase activity, which is directly related to the production of a gene isoform (Cs-HA2). The upregulation of these isoforms is strongly correlated with an increase in the root-to-shoot mobility of activated forms of cytokinins and nitrates (Mora et al., 2010; Gao et al., 2012). The root-to-shoot translocation of both micro- and macro-mineral nutrients also increased due to the signaling effect of nitrate. This effect is associated with cytokinin sink action in the shoot system (Mora et al., 2010; Ramos et al., 2015).

Humic acid triggers an increase in the levels of IAA and NO, which promote shoot growth action; ethylene is also generated in roots, but remains inactive (Mora et al., 2012, 2014). In roots, the application of HS elicits the NO-IAA signaling pathway, which increases the concentration of ABA (Mora et al., 2014). Moreover, high concentrations of sedimentary HS in the rhizosphere reduce hydraulic conductivity in roots, thus, inhibiting plant shoot growth and blocking water uptake by roots (Hose et al., 2000). This is attributed to the accumulation of HA in the pores of the cell wall at the root surface (Asli and Neumann, 2010). Under some experimental scenarios, it was observed that the root hydraulic conductivity is regulated by ABA, where it affects the aquaporin activity of plasma membrane directly (Hose et al., 2000). Therefore, it can be concluded that aquaporin activity and water uptake by the roots is controlled by the rhizospheric concentrations of HS.

The use of ABA-inhibitor fluoridone in combination with HS inhibits root growth, root hydraulic conductivity, and shoot growth. In general, the beneficial impact of HS on shoot development is functionally attributed to IAA, NO, and ABA signaling pathways (Mora et al., 2010). Moreover, these agents regulate the activity of PM H^+^-ATPase via proteins (Takahashi et al., 2012). It can be speculated that interconnected hormone-mediated signaling pathways and the PM H^+^-ATPase pathway together regulate the beneficial actions of HS on shoot growth. However, it is a striking fact that HAs obtained from both vegetal and sedimentary sources share the common domains of signaling pathways in roots (Canellas and Olivares, 2014). The significant impact of both kinds of HS on shoot development can be attributed to their similarity.

To date, numerous studies have been conducted to investigate the effects of HS on root and shoot development, but reports on signaling dynamics with a systems biology approach are scarce and limited. Consequently, more studies are required to further explore this concept. Following the cue, recent reports have demonstrated that the interconnected signaling networks triggered by HS in root and shoot cells likely include ROS signaling pathways (García et al., 2012). HAs derived from vermi-composted vegetal residues are not only able to regulate root development in rice, but also modulate the ROS homeostasis (García et al., 2016a). These properties are connected with transcriptional and post-transcriptional events that modulate the enzymatic activity during oxidative metabolism via their coordinated actions (Takahashi et al., 2012). Consequently, these findings are not surprising, as several studies have unraveled the signaling functions of primary and secondary ROS. As a secondary messenger, ROS mediates root development and architecture, as well as plant responses against biotic and abiotic stresses, by inducing hormonal action through NO and ABA (Cordeiro et al., 2011b). This study speculates that the production and accumulation of ROS is due to HA, and that an antioxidative enzyme network is needed for modulating the final concentration of ROS (Cordeiro et al., 2011a). Lateral root proliferation is supported by the signaling role of ROS, as there is a crucial role played by HA-mediated balance between ROS production and ROS scavenging. Interestingly, an association between HA-mediated effect on ROS homeostasis and root dry weight was reported for rice roots; however, an increase in the hormonal concentrations of IAA, NO, or ETH does not explain this effect (Mora et al., 2012; García et al., 2014; Olaetxea et al., 2016). These studies describe the involvement of two pathways, i.e., hormone-dependent and ROS-dependent effects of HS activity on the root phenotype.

Studies on cucumber sedimentary humic acid obtained from leonardite have also shown that cytokinin and root-to-shoot translocation of mineral nutrients play an important role during shoot growth and development (Mora et al., 2010; Orsi, 2014); however, this effect was associated with an increase in PM H^+^-ATPase activity and root-to-shoot translocation of nitrate (Mora et al., 2010, 2014). Significant improvement in photosynthetic pathways has been reported in rapeseed upon root application of an SHA obtained from black peat (Jannin et al., 2012). A consistent pattern was found among the physiological effects of SHA, thus upregulating the gene clusters controlling all of the above-mentioned physiological pathways such as photosynthesis, CK signaling perception, and N, S, and C metabolisms (Jannin et al., 2012). The shoot growth-promoting action of SHA in cucumber was found to be dependent on the increase in IAA and NO concentrations in roots caused by SHA root application (Mora et al., 2014).

Receptors located in the cell membrane can detect HS, which in turn increases the intracellular concentrations of NO, H_2_O_2_, and H_2_S (Jannin et al., 2012). These elevated concentrations initiate three pathways via post-translational, post-transcriptional, and gene-regulatory modifications, which trigger various homeostatic mechanisms in the plant cell for accelerating the rate of photosynthesis (Orsi, 2014). Moreover, HS upregulate the genes involved in the Calvin cycle, such as genes coding for RUBPCs, G3PdHs, and GPATs, thus increasing starch biosynthesis by stimulating photosynthetic activity (**Figure 2**).

### Humic Substances: Metabolic Activities and Distribution in Tissues

Humic substance plays a vital role in establishing biotic and abiotic interactions within the plant rhizosphere (Kulikova et al., 2014, 2016). However, a comprehensive understanding of the mode of action and tissue distribution of HS is still needed, as this knowledge could then be applied for devising advanced rhizospheric management practices (Kulikova et al., 2016). This section focuses on a discussion of various regulatory activities of HS and its distribution in plant tissues.

Current research on HS is focused on their specific role in metabolic reactions of plants at targeted sites (**Table 3**). Many enzymes have been recognized that participate in different plant responses toward HS, such as Fe (III) chelate-reductase (Kulikova et al., 2014), H^+^-pyrophosphatase (Zancani et al., 2009), plasma membrane H^+^-ATPase (Quaggiotti et al., 2004), tricarboxylic acid cycle enzymes (Kulikova et al., 2016), and glycolytic enzymes (Canellas et al., 2015). It is concluded that HS have multiple regulating functions in plants (**Table 3**), including direct root growth stimulation and proliferation of root hair, maintenance of ion-uptake rates, regulation of the release of protons, redox reactions, and modulation of root exudates (Trevisan et al., 2010; Canellas et al., 2015).

**Table 3:** Humic substance regulating activities in different crops.

Humic substances	Enhanced activities	Crop	Target site	Functions	Reference
HA, FA, and water-soluble fractions	Xanthine and xanthine-oxidase system	*Zea mays*	Leaves, flowers	Defense mechanisms against biotic and abiotic stress from heavy metals	Cordeiro et al., 2011b; García et al., 2016a
HA from manure vermi-compost	Tonoplast aquaporin genes (*OsTIPs*)	*Oryza sativa*	Leaves	Lipid peroxidation and nitrogen metabolism	García et al., 2012
FA	Auxin signaling and homeostasis, H^+^-ATPase, NADPH oxidases, and MPK3	*Arabidopsis thaliana*	Roots	Membrane hyperpolarization, activation of Ca^2+^ channels, and intracellular signaling to increase the growth of secondary roots	Blomster et al., 2011
HA, FA, and HU	Chlorophyllases (a) and (b)	*Pachira macrocarpa*	Leaves	Stimulate the photosynthetic activity and augment the chlorophyll content and conductance of mesophyll cells	Haghighi et al., 2012
HA	Chlorophyllases (a) and (b)	*Lactuca sativa* L.	Leaves	Stimulate the photosynthetic activity and augment the chlorophyll content	García et al., 2012; Haghighi et al., 2012
HA, FA	Phosphoenolpyruvate carboxykinase (PEPC), glutamate dehydrogenase (GDH), MDH, and glutamine synthetase (GS)	*Pinus nigra*	Fruit, leaves and flowers	Activation of carbon and nitrogen metabolism	Jannin et al., 2012; Olaetxea et al., 2015
HU and HA	Phenylalanine ammonia lyase (PAL) and tyrosine ammonia lyase (TAL)	*Brassica napus*	Flowers, roots, and leaves	Triggers nitrogen, sulfur and carbon metabolism	García et al., 2012
HU and FA	Phospholipase A 2, ABA	*Pisum sativum*	Leaves	Regulate stomatal opening	Huang et al., 2013
FA, HA	Peroxidases	*Phaseolus vulgaris* L.		Regulation of plant growth and development, as well as the assimilation of mineral elements such as phosphorus and nitrogen	Aydin et al., 2012
HU, FA, HA	Peroxidase, ABA	*Lycopersicon esculentum* L.	Fruit, leaves and roots	Rise in proline content, protection from hydraulic stress, improvement in growth and development of the plants, even under drought conditions	Hernández et al., 2012; Ramos et al., 2015

Further research is however needed to explain the primary actions of HS. A transcriptomic technique was used to study the primary mode of action of HS (Trevisan et al., 2010, 2011). The expression of 133 genes primarily responsible for transport and catalytic activities, as well as for binding processes, is affected by HS in *Arabidopsis thaliana* (García et al., 2016b). Researchers assumed that HS affect plant growth and other processes via their involvement in the transcription of genes responsible for the formation and organization of the meristem, and organization of microtubules, cytokinesis, and the cell cycle. For this purpose, a directed focus on the entry of HS in roots, their internal transport, and spatial distribution within the plant parts is needed.

A previous study assessed the uptake of HS in plants using synthetic HA with a 14-C label (Vaughan and Ord, 1981). This study concluded that both low- and high-weight fractions of HS can be translocated by plants, but the low-weight fraction showed more penetration than the high-weight HS. This was confirmed by testing the effect of HS conjugated with fluorescein isothiocyanate (FITC) in cultured carrot cells (Muscolo et al., 2007). It was observed that only light-weight HS can be transported across the plasma membrane of carrot cells. In recent studies, tritium-labeled HS have been used to demonstrate the accumulation of labeled material in the roots of wheat seedlings, and its translocation into the shoot (Kulikova et al., 2014). The lipid portion of the treated seedlings was extracted and analyzed, and the tritium label was found in the neutral part of the lipids consisting of alkenes and alkanes that are generally present in waxes and linked with suberized and cuticle tissues. The direct effect of HS on the biosynthesis of lipids in plants, however, remains unclear.

### Humic Substances: Priming Symbiosis and Nodulation

It is widely accepted that HS can accelerate the germination of seeds (Traversa et al., 2013), stimulate ATPase activity, and increase the utilization of nutrients by plants; moreover, HS can also mimic auxin-like hormone activity to promote the growth of plants (Canellas et al., 2010; Jannin et al., 2012). It is a well-accepted fact that the low quality of soil carbon limits the amount of energy available for soil microorganisms. In this case, priming the soil with HS causes an increase in microbial activity due increased availability of energy required by microbes. However, the mechanisms underlying such priming effects are much more complex than commonly believed. Tilba and Sinegovskaya (2012) reported that priming of soybean seeds (under field conditions) with rhizobia, sodium humate, ammonium molybdate, and sodium humate leaf spraying, significantly increased the efficiency of nitrogen fixation and the number of nodules, resulting in an increase in crop yield of up to 22%, compared with the control. Dong et al. (2009) reported that the application of HAs increased the yield of soybean by up to 26.4% and enhanced bacterial diversity, which was further promoted by urea application in the soil. The exact mechanism of the contribution of HAs toward the nitrogen fixation process is still ambiguous. Thus, this review provides a conceptual model of the effects of HS as a priming agent.

Many studies have established that HS can increase the number of rhizobia exponentially to stimulate nitrogen fixation in the soil, thus greatly benefiting sustainable agriculture. Moreover, it is also known that symbiotic rhizobia associations in agricultural systems could lead to the generation of 40 million tons of nitrogen each year (Udvardi and Poole, 2013). Gao et al. (2015) reported that the differential protein expression of soybean in response to water-soluble HS was related to energy production, nucleic acid metabolism, carbon metabolism, and some transmembrane transportation. In addition, HS were found to be more effective than genistein in triggering the expression of nod genes (Ahmad et al., 2012). Canellas et al. (2013) reported that HAs modulate metabolic processes in plants, such as enhancement of PM H^+^-ATPase activity, alteration of sugar and N metabolism, and also induce greater net photosynthesis. Moreover, they reported an increase in the number of rhizobia when inoculation was performed in the presence of soluble HS.

## Conclusion

Humic substances are plant tonic, and modulate the physiological and biochemical processes of plants by triggering multiple interconnected signaling pathways. Although the morphological effects of HS on plants have been comprehensively investigated, the molecular and biochemical basis of these effects still warrant further research. Crops treated with HS are highly tolerant to environmental stresses. To detect HS-plant interactions, priority should be given to increasing our understanding of functional mechanisms that render the highest impact on abiotic stress protection. HS establish transcriptional interactions with biochemical components and signaling pathways, eliciting dynamic signaling crosstalk inside the plant to cope with various types of stresses (Pecinka et al., 2010). Therefore, modeling the role of HS in plants could be a useful tool in filling this knowledge gap. Moreover, HS alleviate the risks of retroelements and genetic instability due to heavy metal stress in plants, and this feature could be a new target for future research. Significant phenotypic effects have been observed in the roots of plants upon HS application at both micro- and macro-morphologic levels (Ramos et al., 2015). Although some reports failed to explain the macro-morphological effects of HS in controlling hormonal increase in plants, these effects are expected to be associated with hormonal regulation, i.e., an increase in IAA, ethylene (ETH), NO, and ABA concentrations in roots (García et al., 2016b). On the other hand, HS are believed to play a vital role in the nodulation process, but questions remain as to their specific role(s), as no comprehensive data are as yet available. Researchers have used whole plants for analyses, instead of specific parts or tissues of plants, and therefore, specific detailed knowledge of the impacts of HS on plant physiology is yet to be gained (Matas et al., 2011). As a result, data on the spatial distribution of HS in the tissues and cells of plants, obtained through genetic studies, would be quite valuable.

## Author Contributions

ZHS and HMR generated the idea for this paper and wrote the manuscript, while HA, BTH, TM, TA, HASA, and YAZ searched the literature. ID and SA critically reviewed, while SY and GC proofread the manuscript.

## Conflict of Interest Statement

The authors declare that the research was conducted in the absence of any commercial or financial relationships that could be construed as a potential conflict of interest.
